# PSO tuned interval type-2 fuzzy logic for load frequency control of two-area multi-source interconnected power system

**DOI:** 10.1038/s41598-023-35454-4

**Published:** 2023-05-30

**Authors:** Ahmed Mohammed Attiya Soliman, Mostafa Bahaa, Mohammed A. Mehanna

**Affiliations:** Department of Electrical Engineering, Faculty of Engineering, Al-Azhar University, Nasr City, Cairo, 1427 Egypt

**Keywords:** Energy science and technology, Engineering

## Abstract

Nowadays, most of modern power systems integrate concentrated renewable energy resources power plants like solar and wind parks in addition to central conventional plants. The output power from these concentrated renewable energy resources varies continuously according to weather conditions like solar irradiance value or wind speed and direction, the variation for their output power may be in mega watts. In this work, Robust secondary load frequency controller (LFC) based on one of artificial intelligent technique which called interval type-2 fuzzy logic controller (IT2FLC) has been proposed for two-area multi-source interconnected power system with central solar park power plants in each area while considering non-linearities in the power system. IT2FLC has accommodated vagueness, distortions and imprecision for the power system input signals which caused by weather fluctuations and system non-linearities. In addition to LFC, another controller based also on IT2FLC has been proposed to control the output power from the central solar parks in each area of generation during cloudy periods instead of maximum power point tracking method (MPPT) in order to enhance the stability for the power system during disturbance periods. In order to enhance the performance of the proposed LFC, particle swarm optimization technique (PSO) has been utilized to optimize the proposed LFC gains to minimize the steady state error, over/under shooting value, settling time and system oscillation for the investigated power system frequency. The performance and the superiority of the proposed PSO tuned IT2FLC is evaluated and compared with another LFC based on PSO tuned cascaded PID controller while applying severe demand load and solar irradiance changes. the simulation has been carried out using matlab/simulink program.

## Introduction

### Problem statement

Most of recent smart grids utilize large scale renewable energy resources like utility scale photovoltaic power plants which known also as solar parks, these solar parks are centralized and supplying the power at the utility level in several megawatts^1,2^ in contrast to distributed generation buildings rooftop mounted PV panels which produces small scale power on the demand level and limited in size^3,4^.the power produced from solar parks are characterized as large output power but also solar irradiance dependant power which varies sharply during cloudy periods or weather fluctuations^5,6^.

On the other hand at the demand side, smart grids large demand loads like public electric vehicles (EV) charging stations have been increased which represent almost 5% of the total united states demand load^7^. These EV charging stations loads are characterized as large, variable and unpredictable demand load^8^.

From all of aforementioned smart grids problems on both utility and demand sides, the power system frequency in smart grids is disturbed from severe changes in utility level generated power due to integration of solar parks as well as severe demand load changes due to increasing the number of EV charging stations.

### Literature review

Several studies have been carried out in order to propose robust load frequency controller for interconnected power systems. These studies have proposed classical controllers like PI, PID and cascaded PID controllers but another studies have proposed modern controllers based on artificial intelligent techniques like fuzzy logic system and neural networks to completely replace classical controllers method while others have proposed hybrid controllers which combine classical and modern controller methods like gain scheduled adaptive controllers, fuzzy PI and fuzzy PID controllers. In^9^, PSO tuned PID controller has been applied to as frequency controller for two area interconnected power system where the proposed controller has achieved less overshoot peak and settling time compared with conventional PI and PID controllers for different scenarios for demand load changes. In^10^, firefly algorithm (FA) tuned 2DOF-PID controller has been proposed as load frequency controller for two area power system where simulation for two different scenarios of changing the demand load in each generating area have been carried out in order to prove the superiority of the proposed controller over FA tuned PID controller. A novel approach has been proposed in^11^ by adding proportional gain for the inner feedback of PID controller which called PID-P controller to be LFC for two-area multi-source power system while the gains of the proposed controller have been optimized using PSO technique, the superiority of the proposed controller has been investigated compared with other controllers like genetic algorithm (GA) tuned PID controller and 2DOF-PID controller. In^12^, PID gains have been tuned using lozi map-based chaotic optimization algorithm (LCOA) for a new proposed objective function, a comparative study has been carried out between the proposed controller and other techniques for optimizing PID controller like GA, PSO and simulated annealing (SA) where the proposed technique has provided better performance than other techniques. Type-1 fuzzy logic controller (T1FLC) has been proposed in^13^ as main LFC for two-area power system integrating solar park power plant and reduction oxidation flow battery (RFB) as fast active power source during disturbance, the proposed controller has achiever better performance compared to PID controller during severe demand load and solar irradiance changes. In^14^, T1FLC has been proposed to act as LFC for two-area power system with interline power flow controller (IPFC) while the gains for the proposed controller have been controlled by PI controller, the proposed controller as well as IPFC have contributed to enhance the power system stability and reducing the system oscillation. Whale optimization algorithm (WOA) tuned IT2FLC has been proposed in^15^ for two-area power system with thyristor controlled phase shifter (TCPS) in tie line as power flow controller, the proposed controller has enhanced the dynamic performance for the power system compared to T1FLC because IT2FLC has handled the uncertainty in the feedback signal. In^16^, artificial bee colony algorithm tuned T1FLC has been proposed in order to control the reserve active power from wind turbine during demand load changes in micro grid power system where the reserve active power is the difference between the wind turbine maximum output power and the de-loaded turbine power while the proposed controller has enhanced the dynamic performance for the turbine rotor speed, pitch angle behavior and the overall micro grid system frequency. Optimal adaptive IT1FLC has been proposed in^17^ to control the de-loaded output power from PV panel in micro grid power system during severe demand load perturbations while the robustness for the proposed controller has been investigated against power system parameters uncertainties, the proposed controller has significantly enhanced the frequency response by decreasing the frequency deviation as well as system settling time.

### Research gaps

In order to stabilize the power system frequency due to disturbances generated in utility or supply side, robust load frequency controller shall be utilized in the power system to control the generation from conventional power plants as well as controlling the output power from renewable energy resources. This controller shall achieve the following:

1. Regulating the output power generated from central conventional power plants like thermal, hydroelectric and gas stations while considering non-linearities in its model such as generation rate constraint (GRC) and governors dead band (GDB).
2. Preserve on frequency deviation levels within controller standard performance (CSP) limits^18^.
3. Maintain the interchange power (tie line power) between generating areas within the pre-scheduled values.
4. Control the output power from the solar park in each area of generation during cloudy periods in order to stabilize the power system during these periods.
5. Maintains the power system stability against severe demand load changes like demand of EV charger stations or large demand loads power outage due to failure in transmission lines.

Most of Classical controllers like (PI, PID or cascaded PID) cannot handle the system frequency problems in modern power systems without continues adaptive tuning for its gains due to several reasons like controller input signals imprecision in modern power systems, power system parameters uncertainties and non-linearities in theses power systems like GRC and GDB.

### Contribution

**Figure 1:**
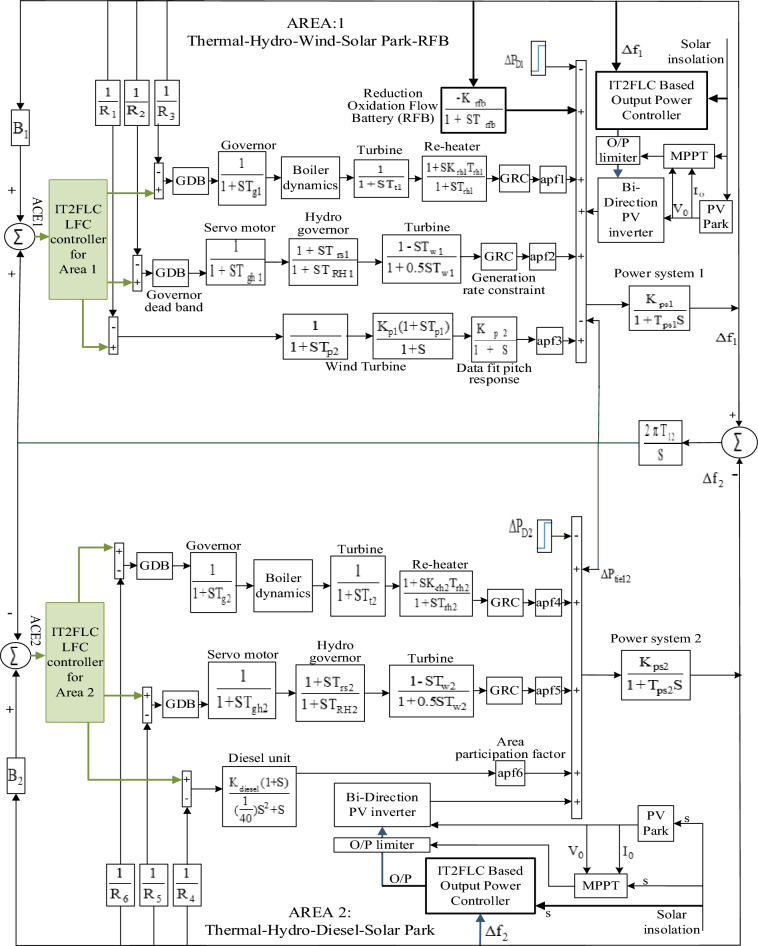
Two-area multi-source investigated power system.

In this work IT2FLC has been proposed as LFC for two-area interconnected power system without power flow controller in tie-line as shown in Fig. 1 while another IT2FLC has been proposed to control the output power from solar park station during cloudy disturbance periods. IT2FLC is one of artificial intelligent techniques which can be utilized as power systems LFC. There are many reasons which make IT2FLC superior to classical controllers such as:

1. It is robust controller where precision of inputs are not necessary^15^.
2. In general, fuzzy logic systems are able to solve complex problems because they resemble human thinking^13^.
3. IT2FLC can handle uncertainties and distortions in input signals better than T1FLC^19^.
4. Flexibility and simplicity of modifying the controller structure and performance by adding or deleting rule base^20^.

As one of the best optimization techniques which has been used with LFC problems^11,13,15^, PSO has been proposed in this work to optimize IT2FLC gains in order to enhance the performance of the proposed controller. PSO is a meta heuristic technique inspired by the movement of swarms like birds or schooling fish while searching for food^21^. This technique optimizes the solution of a problem by assuming a certain number of candidate solutions which called also particles, these particles move according to mathematical function around the search space while each particle changes its position and velocity towards the best swarm or global position for each iteration of searching^22^. For small dimension search spaces like optimizing the gains of the proposed controller in this work, PSO has the advantage over other meta-heuristic optimization techniques like GA, WOA or GWO where it converges fast towards the global best solution while tuning a few number of parameters^23^.

The superiority of the proposed controller will be investigated compared with the performance of PSO tuned PID-P controller proposed in^11^ for the same investigated power system shown in Fig. 1 while applying severe changes for the demand load and solar irradiance levels. The proposed controller performance enhancement due to adding fast active power source like RFB will be investigated in the simulation section compared with the performance of the system without frequency stabilizer.

#### Two-area multi-source investigated power system model

The investigated two-area multi-source power system has been introduced in^11,13,15^. The power system has two generating areas, each area of generation has several generating units. Area-1 has re-heat steam, hydro-electric and wind power plants respectively while area-2 has re-heat steam, hydro-electric and diesel power plants respectively. RFB as frequency stabilizer has been sized for the investigated power system in^13^ and centered in area-1 in order to absorb or discharge power instantaneously during disturbance periods caused by demand load or solar irradiance severe changes because the typical time constant for RFB is only 0.5 ms.^24^. The two generating areas are interconnected through tie-line. In addition to the main task for the proposed LFC to control the power system frequency, the tie-line in the investigated power system does not have any power flow controller which increases the importance of LFC robustness in order to preserve the tie-line power shared between generating areas within its pre-scheduled values. Solar park power plant has been integrated in each generating area with active power capacity of 10% from the power system base power. During normal operation conditions, maximum power point tracker (MPPT) is controlling the solar park main inverter.

In order to maximize the output power from the solar park. In contrast, maximizing the output power from the solar park during disturbances or cloudy periods may affect the power system stability. Consequently in^13^ and^15^, solar park controllers based on T1FLC and IT2FLC respectively has been proposed to control the solar park output power during disturbance periods caused either by severe demand load changes or solar irradiance changes. The proposed solar park controller in^15^ based on IT2FLC has been utilized in this work to control solar park power plants which integrated in area-1 and area-2.

#### Analysis for proposed LFC problems for the investigated power system

Referring to Fig. 1, any changes in area-1 demand load ($P_{D1}$) or area-2 demand load ($P_{D2}$) or both of them create imbalance between the power system generated power and demand load which consequently effects on the frequency of the overall power system not only the frequency of the area that has this change in the demand load because of the tie-line which interconnects the power system generating areas.

The area control error ($ACE$) is defined as the amount of the generated power required to be increased or decreased from a certain area in order to keep the balance between the generated power and the demand load. $ACE$ for the investigated power system can be mathematically expressed as in Eqs. (1,2) for the area control error in area-1 and area-2 respectively:

1 $$ACE_{1} = \Delta P_{tie12} + B_{1} \Delta f_{1}$$

2 $$ACE_{2} = B_{2} \Delta f_{2} - \Delta P_{tie12}$$

where $B_{1}$ and $B_{2}$ are frequency bias sensor for area-1 and area-2 respectively while $\Delta P_{tie12}$ is the shared power through the tie-line between generating areas.

The imbalance between generation and demand in power systems which integrates renewable resources may be also caused by the changes in the generated power from these renewable resources like solar parks, both changes in demand load or generated power create $ACE$ in both areas. The proposed LFC in each area is responsible for increasing or decreasing the generated power in its area while achieving the following objectives:

1. Reducing transient periods settling time.
2. Minimize over/under shooting for the system frequency.
3. Minimize the power system frequency steady state error.
4. Keep the tie-line power within its pre-scheduled values.

The non-linearities in the investigated power system like GDB and GRC in conventional power plants like thermal and hydro-electric stations affect the precision of $ACE$ values which in turn create uncertainties in the input signal for the proposed controller. These uncertainties may affect the stability of the power system and effect on the performance of LFC. IT2FLC in this work takes into consideration these levels of uncertainties in the input signal which enhance the performance IT2FLC compared with T1FLC^19^.

#### Structure of the proposed IT2FLC for LFC in area-1 and area-2

**Figure 2:**
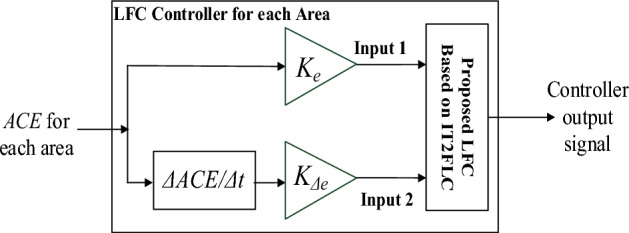
Simplified structure of centralized IT2FLC for LFC in area-1 and area-2.

The proposed LFC is centralized with identical structure in each area of generation as shown in Fig. 2

**Figure 3:**
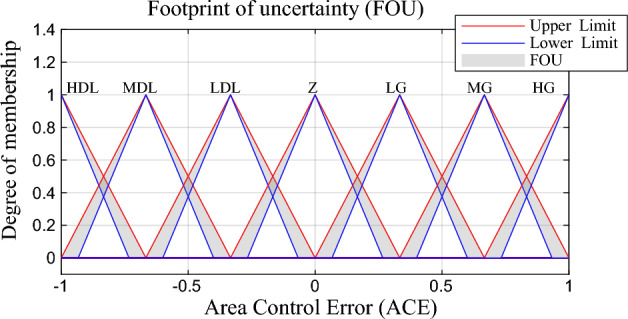
Proposed $ACE$ membership function.

The proposed controller in each area has two inputs which are $ACE$ and the change in the area control error ($\Delta ACE$). $\Delta ACE$ is considered in the proposed controller in order to minimize the level of the oscillation in the power system frequency. The controller input signals are logically supposed to be in the range of (− 1:1) P.U which mean that the control signal from LFC to the power plants shall not exceed 100% (1 P.U) from the rated power system active power capacity while the minimum value of reducing the generation capacity from power plants shall not be less than 100% (− 1 P.U) from the rated power system active power capacity. Any values less or more than (− 1:1) are distortion signals which may occur in transient periods. These distortions may cause malfunction to LFC and consequently instability for the power system, the scaling factors $K_{e}$ and $K_{\Delta e}$ are multiplied by the input signals $ACE$ and $\Delta ACE$ respectively in order to normalize the inputs between (-1:1). The scaled $ACE$ has been classified into seven categories triangle membership functions depending on the kind of the disturbance in the power system as shown in Fig. 3.

Where HDL denotes to high increase in the demand load, MDL is medium increase in the demand load, LDL is low increase in the demand load while Z denotes to no change in the demand load or the power generation in the power system and the system is stable. On the other hand, HG denotes to high increase in the generated power compared with the demand load, MG is medium increase in the generated power while LG denotes to low increase in the generated power compared with the demand load. The uncertainty in $ACE$ signal due to distortion or non-linearities in the power system has been represented in the grey area in the membership function which called foot print of uncertainty level, this area in this work is assumed to be fixed value with 10% of uncertainty.

**Figure 4:**
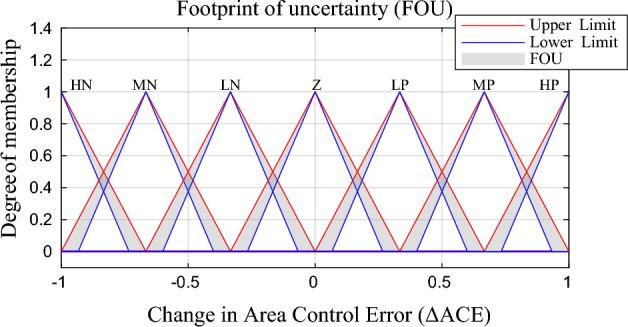
Proposed $\Delta ACE$ membership function.

The scaled $\Delta ACE$ also has been classified into seven categories triangle equal membership functions (high negative, medium negative, low negative, zero, low positive, medium positive and high positive) or (HN, MN, LN, Z, LP, MP, HP) as shown in Fig. 4. The uncertainty in $\Delta ACE$ signal due to system oscillation has been represented in the grey area in the membership function and also assumed to be fixed value with 10% of uncertainty.

The proposed controller output signal to increase or decrease the generation from the power plants depends on the combination of $ACE$ and $\Delta ACE$ linguistic status which can be summarized in the rule base table. The rule base depends mainly on designer reasoning and system experience as shown in the proposed rule base in Table. 1.

**Table 1:** Proposed rule base correlates $ACE$, $\Delta ACE$ and controller output.

Scaled $ACE$	HDL	MDL	LDL	Z	LG	MG	HG
Scaled $\Delta ACE$							
HN	HP	HP	HP	MP	MP	LP	Z
MN	HP	MP	MP	MP	LP	Z	LN
LN	HP	MP	LP	LP	Z	LN	MN
Z	MP	MP	LP	Z	LN	MN	MN
LP	HP	LP	Z	LN	LN	MN	HN
MP	LP	Z	LN	MN	MN	MN	HN
HP	Z	LN	MN	MN	HN	HN	HN

**Figure 5:**
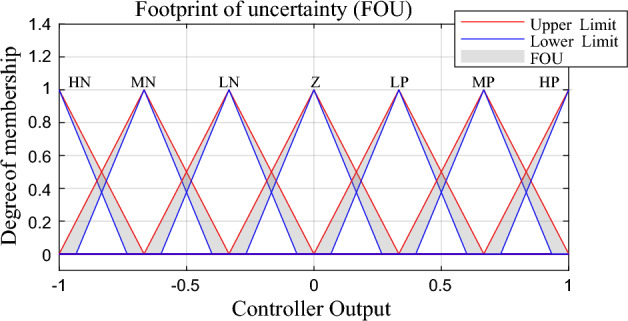
Proposed controller output membership function.

Referring to Table 1, the proposed IT2FLC controller output action is also categorized into seven status (HN, MN, LN, Z, LP, MP, HP). The action required according to the proposed rule base is then applied to the controller output membership function as shown in Fig. 5 where the controller action is converted to digital or crisp value in the range of (-1:1) PU in a process called defuzzificatuion^25^.

#### PSO tuning for the proposed LFC scaling factors ($K_{e}$) and ($K_{\Delta e}$)

In this work, PSO algorithm proposed in^26^ has been utilized to enhance the performance of the proposed LFC. The objective of PSO is tuning the controller scaling factors $K_{e}$ and $K_{\Delta e}$ in order to minimize the overall power system error. The overall power system error has been defined in this work as expressed in Eq. (3).

3 $$OE = \Delta f_{1} + \Delta f_{2} + \Delta P_{tie12}$$

In order to minimize the power system frequency error and system oscillation as well as reduce the system settling time, the multi-objective function proposed in^13,15^ has been suggested in this work in order to evaluate the fitness of each particle in the swarm. The objective function is expressed in Eq. (4) while the simulation time is 60 s.

4 $$Objective\, Function = 0.2\mathop \smallint \limits_{0}^{60\,s} OE^{2} \cdot dt + 0.8 \mathop \smallint \limits_{0}^{60\,s} t \cdot OE^{2} \cdot dt$$

The proposed objective function combines fractionally the features of two different single objective functions which are integral square error (ISE) and integral time square error (ITSE), ISE $\left( {\int_{0}^{60\,s} {OE^{2} \cdot d} } \right)$ tends to reduce over/under shooting values in the power system frequency during transient period because it penalizes large errors more than smaller errors since the square of a large errors will be larger. On the other hand, ITSE $\left( {\int_{0}^{60\,s} {t \cdot OE^{2} \cdot d} } \right)$ tends to minimize the power system frequency steady state error because it penalizes small errors more than large errors by multiplying the small errors by the time. Evaluation of the proposed objective function and its superiority over single objective functions has been introduced in details in^13^. The fraction value for each single objective functions determines the objective from the optimization technique which also depends on system experience.

**Figure 6:**
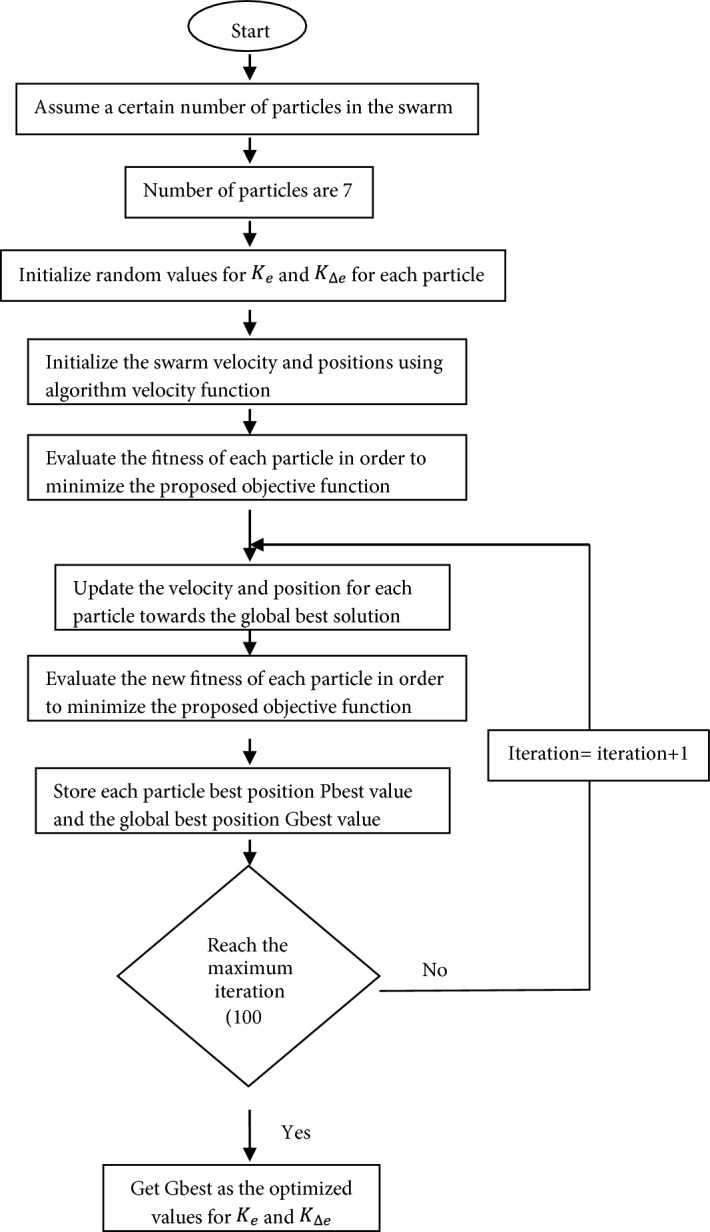
PSO flow chart for optimizing LFC scaling factors.

The flow chart shown in Fig. 6 illustrates the steps of PSO algorithm for optimizing the scaling factors of the proposed LFC.

**Figure 7:**
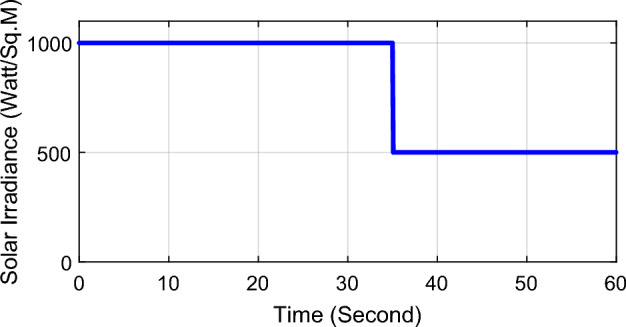
Solar irradiance pattern from high irradiance to low at t = 35 s.

PSO algorithm has been run in order to optimize the scaling factors for the proposed LFC in the investigated power system using matlab/simulink program as per the steps shown in Fig. 6 while applying 10% step demand load change in area-1 and solar irradiance pattern as shown in Fig. 7. For 100 iterations, the global best values for LFC scaling factors are $K_{e} = 38.5$ and $K_{\Delta e} = 22.5$. The value of the scaling factors obtained will be used in the next section of simulation and results in order to investigate the robustness of the proposed controller for several power system disturbances without retuning the controller gains.

## Simulation and results

**Figure 8:**
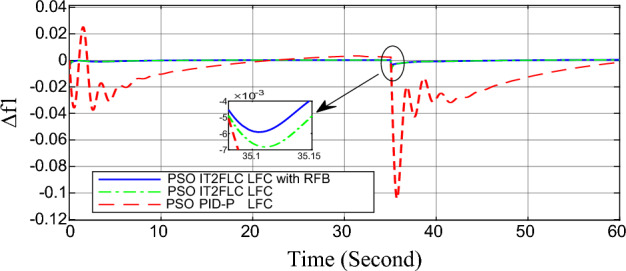
First case of study change in area-1 frequency ($\Delta f_{1}$).

**Figure 9:**
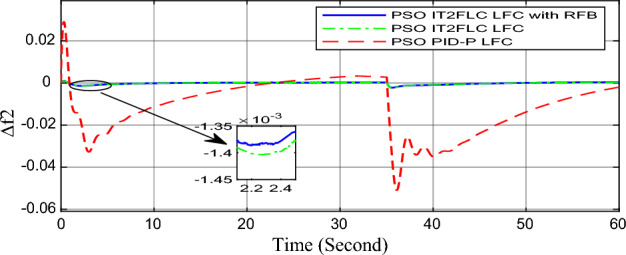
First case of study change in area-2 frequency ($\Delta f_{2}$).

**Figure 10:**
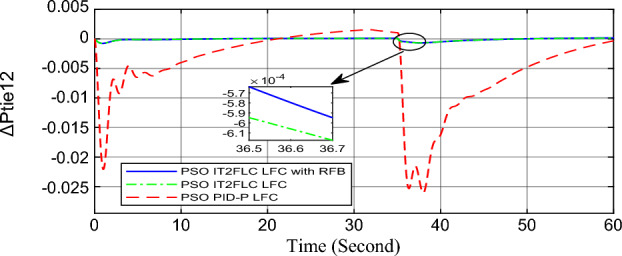
First case of study change in tie-line scheduled power ($\Delta P_{tie2}$).

**Figure 11:**
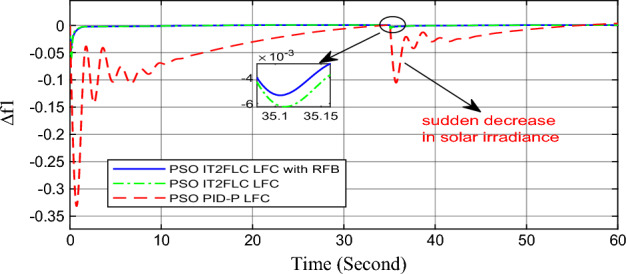
Second case of study change in area-1 frequency ($\Delta f_{1}$).

**Figure 12:**
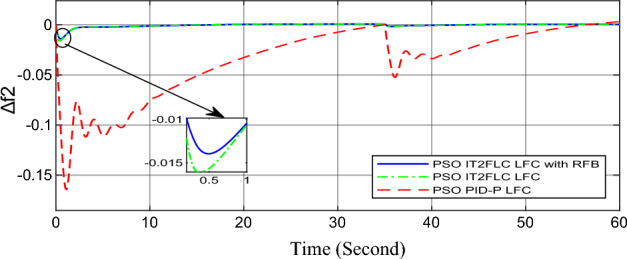
Second case of study change in area-2 frequency ($\Delta f_{2}$).

**Figure 13:**
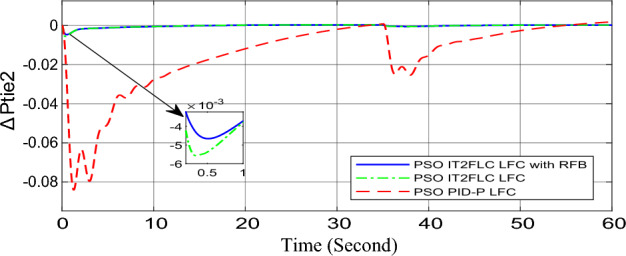
Second case of study change in tie-line scheduled power ($\Delta P_{tie2}$).

**Figure 14:**
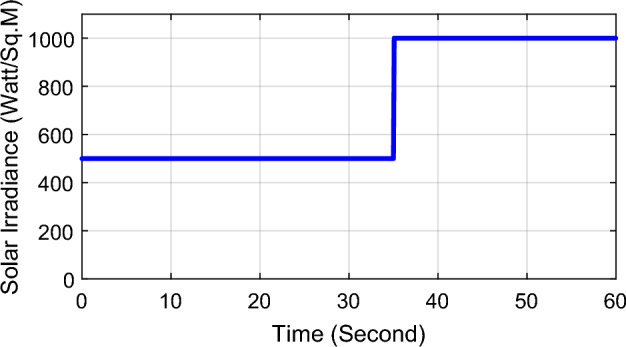
Solar irradiance pattern from low irradiance to high at t = 35 s.

**Figure 15:**
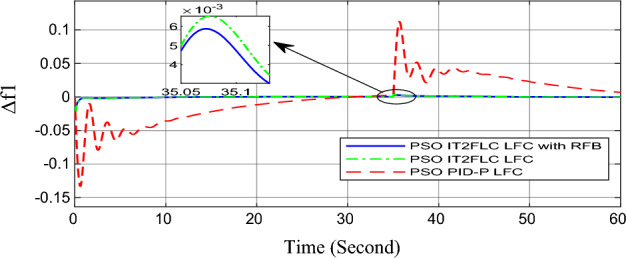
Third case of study change in area-1 frequency ($\Delta f_{1}$).

**Figure 16:**
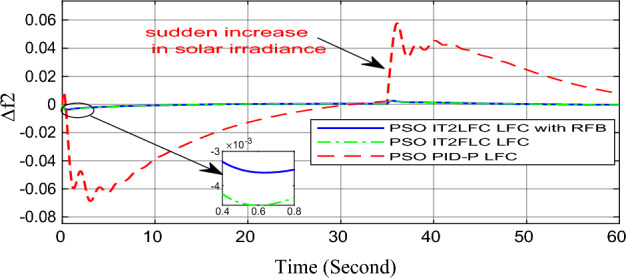
Third case of study change in area-2 frequency ($\Delta f_{2}$).

**Figure 17:**
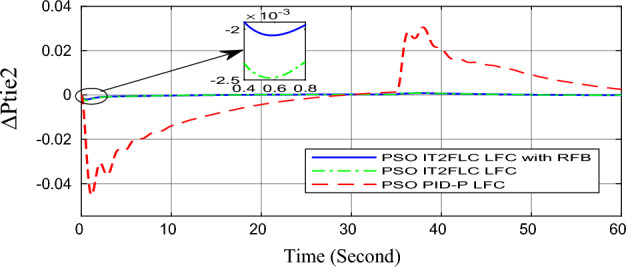
Third case of study change in tie-line scheduled power ($\Delta P_{tie2}$).

**Figure 18:**
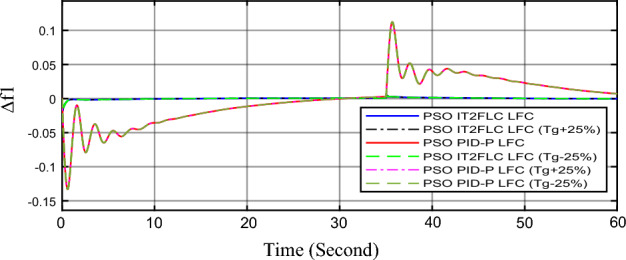
Fourth case of study change in area-1 frequency ($\Delta f_{1}$) for different speed governor time constant values.

**Figure 19:**
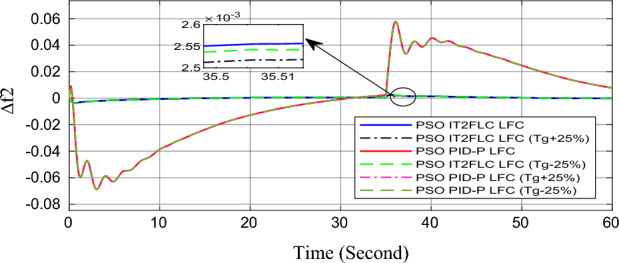
Fourth case of study change in area-2 frequency ($\Delta f_{2}$) for different speed governor time constant values.

**Figure 20:**
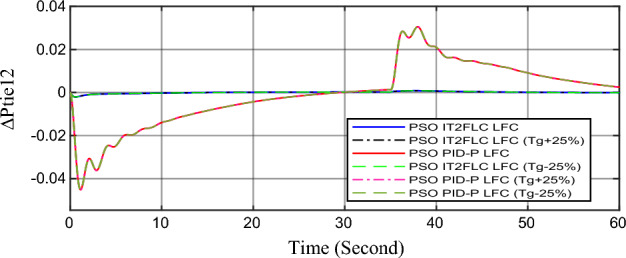
Fourth case of study change in tie-line scheduled power ($\Delta P_{tie2}$) for different speed governor time constant values.

**Figure 21:**
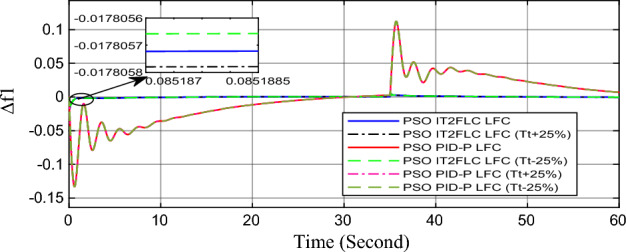
Fourth case of study change in area-1 frequency ($\Delta f_{1}$) for different steam turbine time constant values.

**Figure 22:**
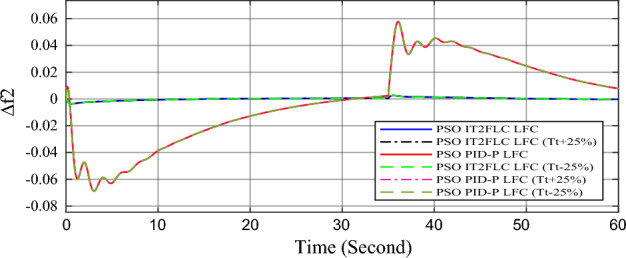
Fourth case of study change in area-2 frequency ($\Delta f_{2}$) for different steam turbine time constant values.

**Figure 23:**
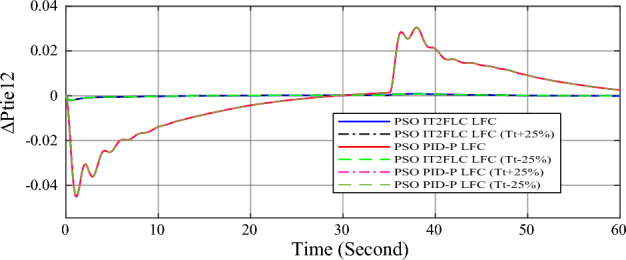
Fourth case of study change in tie-line scheduled power ($\Delta P_{tie2}$) for different steam turbine time constant values.

In this part of the paper, the performance of the proposed LFC based on IT2FLC will be simulated for the power system shown in Fig. 1. The superiority of the proposed controller will be investigated over cascaded PID based controller which has been proposed in^11^ and introduced for comparison in^13^ while applying three different cases of studies. The influence of integrating frequency stabilizer like RFB has been also studied in these cases of studies in order to investigate RFB role for enhancing the performance of the proposed controller. The cases of studies can be described as follows:

1. *First case of study*: In this case of study, step 10% increase has been applied to demand load in area-1 while the solar irradiance pattern is as shown in Fig. 7 (sudden decrease in solar irradiance from 1000 to 500 w/m^2^ at t = 35 s). The results are shown in Figs. 8, 9 and 10 for the frequency deviation in area-1, area-2 and the deviation in tie-line power respectively.
2. *Second case of study*: In this case of study, step 20% increase has been applied to demand load in area-1 and area-2 while the solar irradiance pattern is as shown in Fig. 7. The results are shown in Figs. 11, 12 and 13 for the frequency deviation in area-1, area-2 and deviation in tie-line power respectively.
3. *Third case of study*: In this case of study, 10% increase has been applied to demand load in area-1 while the solar irradiance pattern is as shown in Fig. 14 (sudden increase in solar irradiance from 500 to 1000w/m^2^ at t = 35 s). The results are shown in Figs. 15, 16 and 17 for the frequency deviation in area-1, area-2 and deviation in tie-line power respectively.
4. *Fourth case of study*: uncertainty for the values of the power system parameters like speed governors time constant ($T_{g1}$ and $T_{g2}$) or steam turbine time constant ($T_{t1}$ and $T_{t2}$**) **may cause malfunction for the operation of the proposed controller during practical investigation. Consequently in this case of study, changes (± 25%) in the investigated power system parameters like $T_{g1}$, $T_{g2}$, $T_{t1}$ and $T_{t2}$ have been applied in order to investigate the sensitivity of the proposed controller against power system parameters changes while applying 10% increase has been applied to demand load in area-1 while the solar irradiance pattern is as shown in Fig. 14 (sudden increase in solar irradiance from 500 to 1000 w/m^2^ at t = 35 s). The results are shown in Figs. 18, 19, 20 for the frequency deviation in area-1, area-2 and deviation in tie-line power respectively due to speed governor time constant changes while Figs. 21, 22 and 23 for the frequency deviation in area-1, area-2 and deviation in tie-line power respectively due to steam turbine time constant changes.

In the first and the second cases of studies, the robustness of the proposed controller has been investigated in Figs. 8, 9, 10, 11, 12 and 13. in contrast to cascaded PID-P controller, PSO tuned IT2FLC has reduced significantly the settling time, system oscillation and the steady state error during severe demand load change in area-1, area-2 and severe instantaneous decrease in solar irradiance value which has been occurred simulation period at t = 35 s. The proposed controller has managed to preserve the stability of the investigated power system despite the influence of conventional power plant non-linearities like GRC and GBD. The footprint of uncertainty in the proposed IT2FLC controller has mitigated undesired system oscillation and enhanced the overall stability for the investigated power system. Integration of RFB has enhanced the performance of the proposed controller due to its ability to inject or absorb power in a short time which typically less than 0.5 ms.

On the other hand, the solar irradiance in the third case of study has been raised instantaneously from low level (500 W/m^2^) to its maximum value (1000 W/m^2^) at t = 35 s which caused the power generated in area-1 and area-2 to be increased sharply and consequently instantaneous increase in the investigated power system frequency, the proposed controller has mitigated this increase in system frequency in short time within 0.3 s with overshoot value less than 0.005 P.U. In contrast, cascaded PID controller has settled the system due to this disturbance in more than 9 s while the overshoot value for the frequency has exceeded 0.11P.U. For the power system which integrates RFB, RFB has absorbed this increase in the power generated and as a result RFB has enhanced significantly the performance of the proposed IT2FLC controller.

Finally in the fourth case of study, sensitivity analysis for the proposed controller has been investigated in order to confirm the robustness of the proposed LFC in case of power system parameters uncertainty. As shown in the figures for this case of study, the proposed controller has preserved on the stability of the investigated power system despite the large changes in generation units time constants (± 25%). The steady state error in system frequency as well as settling time has been deviated by (± 0.0025%) from the fundamental curves without retuning for the proposed controller gains.

Numerical analysis for the first, the second and the third cases of studies has been summarized in Table 2 which showing the maximum overshooting (O.S), under shooting (U.S), the power system settling time and the value of steady state error (SSE) for the proposed controller with and without RFB compared with cascaded PID-P controller.

**Table 2:** Numerical analysis for the first, the second and the third cases of studies.

Case of study	Parameter	LFC controller	Settling time (s)	Max. O.S (p u)	Max. U.S (p u)	SSE (p u)
First case of study	$\Delta f_{1}$	PSO IT2FLC LFC with RFB	6.3	0.0001	− 0.0014	0.0001
		PSO IT2FLC LFC	6.9	0.0006	− 0.002	0.0001
		PSO PID-P LFC	13.9	0.0017	− 0.04	0.001
	$\Delta f_{2}$	PSO IT2FLC LFC with RFB	6.8	0.0003	− 0.001	0.0002
		PSO IT2FLC LFC	7.1	0.0007	− 0.003	0.0002
		PSO PID-P LFC	26	0.002	− 0.04	0.001
	$\Delta P_{tie12}$	PSO IT2FLC LFC with RFB	7.2	0.00015	− 0.0005	0.00009
		PSO IT2FLC LFC	7.5	0.0002	− 0.0009	0.00009
		PSO PID-P LFC	25	0.0017	− 0.024	0.0007
Second case of study	$\Delta f_{1}$	PSO IT2FLC LFC with RFB	7.2	0.0003	− 0.0019	0.0002
		PSO IT2FLC LFC	7.9	0.0009	− 0.0035	0.0002
		PSO PID-P LFC	32	0.08	− 0.38	0.019
	$\Delta f_{2}$	PSO IT2FLC LFC with RFB	7.6	0.0005	− 0.005	0.0003
		PSO IT2FLC LFC	8.1	0.001	− 0.008	0.0003
		PSO PID-P LFC	32	0.04	− 0.18	0.019
	$\Delta P_{tie12}$	PSO IT2FLC LFC with RFB	7.9	0.00019	− 0.0009	0.0001
		PSO IT2FLC LFC	8.3	0.0003	− 0.001	0.0001
		PSO PID-P LFC	32	0.027	− 0.098	0.0079
Third case of study	$\Delta f_{1}$	PSO IT2FLC LFC with RFB	6.4	0.0001	− 0.0016	0.0001
		PSO IT2FLC LFC	7	0.0006	− 0.0019	0.0001
		PSO PID-P LFC	13.9	0.0017	− 0.04	0.001
	$\Delta f_{2}$	PSO IT2FLC LFC with RFB	6.2	0.0004	− 0.0015	0.00015
		PSO IT2FLC LFC	6.7	0.0008	− 0.0025	0.00015
		PSO PID-P LFC	26	0.002	− 0.04	0.001
	$\Delta P_{tie12}$	PSO IT2FLC LFC with RFB	6.8	0.0001	− 0.0006	0.0001
		PSO IT2FLC LFC	7.12	0.0003	− 0.0008	0.0001
		PSO PID-P LFC	23	0.0019	− 0.03	0.001

## Conclusions

In this work, IT2FLC has been proposed to act as main LFC for two-area multi-sources interconnected power system integrating solar park power plant in each area. Gains for the proposed controller have been tuned using PSO optimization technique. IT2FLC LFC performance has been investigated and compared to cascaded PID LFC proposed in previous work for several case studies while changing demand load in each generating area and changing the solar irradiance during simulation period to investigate the robustness of the proposed controller. IT2FLC as main LFC has enhanced the stability for the power system by reducing system settling time, over/under shooting and steady state error. The footprint of uncertainty in the proposed controller has significantly reduced undesired system oscillation during transient and steady state periods. Integration of RFB has enhanced the performance of the proposed controller since it has reduced the power system settling time, oscillation and over/under shooting values. In order to extend this work in future works, recent developed meta heuristic optimization techniques like Firefly, moth-flame and slap swarm can be utilized instead of PSO technique in order to tune the controller gains. In future works also, type 3 fuzzy logic system can be developed from IT2FLC in order to enhance the response of LFC especially the power system oscillation in transient period caused by the uncertainty for the input values.

### Investigated power system parameters

${\varvec{K}}_{{{\varvec{RFB}}}} = {{{\varvec{0.6777}}}}$; ${\varvec{T}}_{{{\varvec{RFB}}}} = {{{\varvec{0.00034}}}}$ s; ${{{{\varvec{ T}}}}}_{{{\varvec{g}}1}} { } = {{{{\varvec{ T}}}}}_{{{\varvec{g}}2}} { } = {{{\varvec{{ }0.08}}}}$ s; ${\varvec{T}}_{{{\varvec{t}}1}} { } = {{{{\varvec{ T}}}}}_{{{\varvec{t}}2}} { } = {{{\varvec{0.3}}}}$sS; ${\varvec{K}}_{{{\varvec{r}}1}} { } = {\varvec{K}}_{{{\varvec{r}}2}} { } = {{{\varvec{{ }0.333}}}}{\varvec{Hz}} / {\varvec{p}}.{\varvec{{ u}}}.{\varvec{MW}}$; ${\varvec{T}}_{{{\varvec{r}}1}} { } = {{{\varvec{{ T}}}}}_{{{\varvec{r}}2}} { } = {{{\varvec{{ }10}}}}$ s; ${{{\varvec{{ T}}}}}_{{{\varvec{GH}}1}} { } = {{{\varvec{{ T}}}}}_{{{\varvec{GH}}2}} { } = {{{\varvec{{ }48.7}}}}$ s; ${\varvec{T}}_{{{\varvec{RS}}1}} { } = {{{\varvec{{ T}}}}}_{{{\varvec{RS}}2}} { } = {{{\varvec{{ }0.513}}}}$ s; ${\varvec{T}}_{{{\varvec{RH}}1}} { } = {{{\varvec{{ T}}}}}_{{{\varvec{RH}}2}} { } = {{{\varvec{{ }10}}}}$ s; ${\varvec{T}}_{{{\varvec{W}}1}} { } = {{{\varvec{{ }1}}}}$ s; ${\varvec{K}}_{{{\varvec{p}}1}} { } = {{{\varvec{{ }1.25{ Hz}}}}} / {\varvec{p}}.{{{\varvec{{ u}}}}}.{\varvec{MW}};{{{\varvec{{ T}}}}}_{{{\varvec{p}}1}} { } = {{{\varvec{{ }6{ sec}}}}};{{{\varvec{{ T}}}}}_{{{\varvec{p}}2}} { } = {{{\varvec{{ }0.041{ s}}}}}$; ${\varvec{K}}_{{{\varvec{p}}2}} { } = {{{\varvec{{ }1.4}}}}$; ${\varvec{K}}_{{{\varvec{diesel}}}} { } = {{{\varvec{16.5{\, S}}}}}$; ${\varvec{R}}_{1} = {{{\varvec{{ R}_{2}}}}} = {\varvec{R}}_{3} = {\varvec{R}}_{4} = {\varvec{R}}_{5} = {\varvec{R}}_{6} =$ 2.4 Hz/P.U MW; ${\varvec{B}}_{1} = {{{\varvec{{ B}_{2}}}}} =$ 0.425P.U MW/Hz; ${\varvec{K}}_{{{\varvec{PS}}1}} = {{{\varvec{{ K}}}}}_{{{\varvec{PS}}2}} =$ 120 Hz/P.U MW; ${\varvec{T}}_{{{\varvec{PS}}1}} = {{{\varvec{{ T}}}}}_{{{\varvec{PS}}2}} =$ 20 s; ${\varvec{T}}_{12} =$ 0.08 P.U MW/Hz; Solar park power rating at standard test conditions (STC) = 0.1 P.U.

### Proposed controller parameters

Type of inference system = Mamdani FIS; Number of inputs = 2; Scaling factor for input 1 = 38.5; Scaling factor for input 2 = 22.5; Input 1 range = [0 1]; Input 2 range = [0 1]; Output range = [0 1]; De-fuzzification method = Centroid; Type of inputs and output membership function = equal triangle mf; Number of input 1 mf = 7; Number of input 2 mf = 7; Number of output mf = 7.

### PSO algorithm parameters

Number of variables = 2 (${\varvec{K_{e}}}$ and ${{{\varvec{K_{\Delta e}}}}}$); Cost function = ${{{\varvec{0.2\int_{0}^{60\sec } {OE^{2} \cdot dt} + 0.8 \int_{0}^{60\sec } {t \cdot OE^{2} \cdot dt}}}}}$; Variables minimum value = -10; Variables maximum value = 100; Number of particles = 7; Maximum iterations number = 100; Inertia weight = 1; Acceleration coefficient 1 = 2.05; Acceleration coefficient 1 = 2.05.

## Data Availability

The datasets used and/or analysis during the current study available from the corresponding author on reasonable request.

## Abbreviations

ACE: Area control error
AGC: Automatic generation control
APF: Area participation factor
CSP: Controller standard performance
FACTS: Flexible alternating current transmission system
FLS: Fuzzy logic system
GDB: Governor dead band
GRC: Generation rate constraint
GWO: Grey wolf optimization
IT2FLC: Interval type 2 fuzzy logic controller
LFC: Load frequency controller
MPPT: Maximum power point tracker
PSO: Particle swarm optimization
RFB: Reduction oxidation flow battery
T1FLC: Type-1 fuzzy logic controller
